# Drugs of Addiction

**Published:** 1923-03

**Authors:** 


### " DRUGS OF ADDICTION."

THE world is fond of talking about the horrors of barbarism; but the horrors of civilisation would seem to the reflective mind to be at least as worthy of grave consideration. The higher the type of civilisation the greater the certainty of an outcrop of moral and social evils. They were abundant enough in Greece, where, for the matter of that, they were treated with a certain philosophic toleration, and had its successive forerunners, Babylon and Egypt, left us any literature to speak of, we should probably find that, as they mounted the ladder of what approved itself to their eyes as progress, their sociological difficulties grew more complicated. Civilisation and Empire move together towards the setting sun, and the nearer they approach to it the less able they seem to free themselves of the social dangers which have ever beset the twain. Many of these dangers cannot appropriately be discussed here—they are primarily for the physician of the soul. Yet few indeed are the moral difficulties which have not their reactions upon physical health, and just now the physician and the moralist, who, between them, should constitute the ideal Minister of Health, are faced by a trouble—the addiction to drugs—which has of late become more widespread and insistent than ever.

During the past month Sir William Collins and other distinguished authorities have been making a further attempt to awaken the public conscience to the perils which underlie the indiscriminate use of powerful narcotics—the weakening of the will, the deterioration of the moral fibre, the upsetting of spiritual values, the inevitable, and speedy, destruction of mind and body. The enhanced rapidity of life, the growing pressure of business and pleasure, the clangour and excitement of existence amid great populations, the ever-increasing burden upon body, nerve, and brain—all these predispose the imperfectly balanced to seek new forms of excitement, fresh means of obtaining respite. Drink has become not merely old-fashioned but gross—that method of finding oblivion is *vieux jeu*. The weak-willed and the vicious now fly to the murderous narcotics which the specialist calls " drugs of addiction "—cocaine, morphia, opium, and others less familiar, but hardly less potent. The extent to which these things are consumed is suggested both by the many instances of their fatal effects with which the newspapers have teemed during the last year or two, but by the almost innumerable prosecutions of those who make handsome profits by supplying them to their craving victims. M. Victor Margueritte has defended himself against the well-founded condemnation of his notorious novel, " La Garçonne," by pleading that he wrote it in large measure to bring home to the world the physical and moral horrors of indulgence in drugs as the newest and, in some respects, the worst, development of vice. He would have been more successful had he refrained from the description of scenes and the use of language hitherto to be found only in books which people are sent to prison for selling. Yet with all his exaggerations and his unpardonable lubricities, he has placed his finger upon one of the most insidious and destructive of vices.

It is of very little use to reason with those who are given to the constant and increasing use of narcotic drugs. They have to be saved from themselves and from the pollution they spread around them, and this can only be done by making it impossible for them to obtain the means of ruin. Experience, however, shows that this is exceedingly difficult so long as the production of these drugs vastly exceeds the legitimate demand. The Assembly of the League of Nations has laid it down that the most effective way of stopping a traffic so remunerative that it is worth while to run great risks in its conduct is to control production. Hundreds of thousands of kilogrammes of opium are grown and tons of morphia manufactured annually; yet the medical needs of the world put together are very small. In 1921 ten large London hospitals treated more than 620,000 in- and out-patients, yet they used among them less than forty-two pounds of opium, less than five and a half pounds of morphia, and not much more than nine pounds of cocaine. Nor is there any reason to suppose that the medical requirements of other countries are, relatively, on any larger scale. There would be little difficulty in ascertaining, with approximate accuracy, the world's annual need of drugs which, in the right hands, may give back life, but in the wrong are certain to destroy it, and, in the process, to spread demoralisation to an incalculable extent. If the League of Nations can help effectively to impose control upon the production of these narcotics it will do much to remove one of the worst reproaches with which civilisation has too readily allowed itself to be encrusted. Pending comprehensive international action, the Home Secretary has brought in a Bill to amend the Dangerous Drugs Act, which enormously increases the penalties for traffickers in madness and death.

